# Skeletal Microstructure in Addison's Disease

**DOI:** 10.1210/jendso/bvaf180

**Published:** 2025-11-11

**Authors:** Leonardo Bandeira, Rodrigo Nolasco dos Santos, Gustavo de Paula Ripka, Juan P dos Santos Rossi, Sanchita Agarwal, Claudio E Kater, Flavia A Costa-Barbosa, Marcella D Walker, John P Bilezikian, Marise Lazaretti-Castro

**Affiliations:** Division of Endocrinology, Department of Medicine, Universidade Federal de Sao Paulo, Sao Paulo 04077-020, Brazil; Division of Endocrinology, Department of Medicine, Vagelos College of Physicians and Surgeons, Columbia University, New York, NY 10032, USA; Division of Endocrinology, Department of Medicine, Universidade Federal de Sao Paulo, Sao Paulo 04077-020, Brazil; Division of Endocrinology, Department of Medicine, Universidade Federal de Sao Paulo, Sao Paulo 04077-020, Brazil; Division of Endocrinology, Department of Medicine, Universidade Federal de Sao Paulo, Sao Paulo 04077-020, Brazil; Division of Endocrinology, Department of Medicine, Vagelos College of Physicians and Surgeons, Columbia University, New York, NY 10032, USA; Division of Endocrinology, Department of Medicine, Universidade Federal de Sao Paulo, Sao Paulo 04077-020, Brazil; Division of Endocrinology, Department of Medicine, Universidade Federal de Sao Paulo, Sao Paulo 04077-020, Brazil; Division of Endocrinology, Department of Medicine, Vagelos College of Physicians and Surgeons, Columbia University, New York, NY 10032, USA; Division of Endocrinology, Department of Medicine, Vagelos College of Physicians and Surgeons, Columbia University, New York, NY 10032, USA; Division of Endocrinology, Department of Medicine, Universidade Federal de Sao Paulo, Sao Paulo 04077-020, Brazil

**Keywords:** HRpQCT, bone microarchitecture, Addison's disease, glucocorticoid, skeletal, microstructure

## Abstract

**Context:**

Addison's disease (AD) is characterized by deficient adrenal glucocorticoid (GC) production. Treatment involves just GC replacement, but patients often receive high doses, leading to side effects. Bone mineral density (BMD) data in AD are conflicting. High-resolution peripheral quantitative computed tomography (HRpQCT) evaluates volumetric BMD, microarchitecture, and mechanical properties of the tibia and radius. No studies have assessed bone quality by HRpQCT on subjects with AD.

**Objective:**

Evaluate the bone health of patients with AD using HRpQCT.

**Design:**

Cross-sectional study.

**Setting:**

Ambulatory of a tertiary medical center.

**Participants:**

Nineteen patients with AD on GC were compared to 38 matched controls.

**Main Outcome Measures:**

Dual-energy x-ray absorptiometry and HRpQCT measurements.

**Results:**

Patients with AD had lower lean mass and BMD. At the radius, the AD group had 11% lower trabecular (Tb) number (*P* = .03). At the tibia, patients had lower Tb number and Tb volumetric BMD, greater Tb separation, and lower cortical area and thickness (17-26% difference in these parameters, *P* < .03 for all). Tibial stiffness was 21% lower in the AD group (*P* < .03). In this group, there was a positive correlation between lean mass and stiffness (radius r = 0.53, tibia r = 0.51; both *P* < .04) and a negative correlation between cumulative GC dose and spine BMD (r = −0.67, *P* < .01).

**Conclusion:**

This is the first study to assess bone using HRpQCT in patients with AD on GC. Our findings suggest that AD patients have a loss of lean mass and skeletal fragility, mainly of the trabecular compartment and at the tibia. Bone loss may be related to loss of lean mass and GC.

Primary adrenal insufficiency or Addison´s disease (AD) is a rare life-threatening condition characterized by adrenocortical glucocorticoid (GC) and mineralocorticoid (MC) deficiency. Autoimmune adrenalitis is the most common cause of acquired AD in high-income countries, with a prevalence of approximately 100 cases per million. Age onset is around 30 to 50 years, and women are more affected than men [1-3]. In developing countries, tuberculosis and paracoccidioidomycosis are also important causes of AD [4, 5].

Glucocorticoid and MC replacement therapy for patients with AD is often a challenging task [6]. Available GC formulations fail to replicate the physiological circadian and ultradian rhythmicity of cortisol secretion [7], and the threshold between physiologic and supraphysiologic GC doses is difficult to achieve [6]. Not infrequently, patients are exposed to higher than physiologic GC doses and to long-term side effects, including poor quality of life and high cardiovascular risk [8, 9].

Regarding bone health, there are several mechanisms by which chronic exposure to GC excess may result in skeletal deterioration [10, 11]. Long-term effects include direct inhibition of osteoblasts and, consequently, reduced bone formation. In addition, increased renal calcium excretion and decreased intestinal calcium absorption (due to inhibition of vitamin D's physiological actions to promote calcium absorption) induce a secondary hyperparathyroidism, in which bone resorption may be enhanced at the outset of therapy. Moreover, GC can have a myopathic effect, in which muscle weakness leads to a higher risk of falls, which may result in fractures. Also, in AD, adrenal androgens are also lacking. They may also have reduced hepatic IGF-1 secretion, leading to additional risks [12-14]. There is limited data regarding bone involvement in patients with primary adrenal insufficiency chronically receiving GC replacement.

The recommended method for evaluating bone mineral density (BMD) and for diagnosing osteoporosis is dual-energy x-ray absorptiometry (DXA) [15, 16]. However, DXA depicts an area-based BMD (aBMD) rather than a volumetric BMD (vBMD). Thus, either larger or smaller bones can induce a measurement artifact. Furthermore, DXA provides information about the amount of mineralized bone (density) that does not necessarily reflect bone quality.

High-resolution peripheral quantitative computed tomography (HRpQCT) is a noninvasive in vivo method by which skeletal features not accessible by DXA can be determined, including vBMD, cortical and trabecular microarchitecture, and mechanical properties of the distal tibia and radius [17-19]. Bone strength is estimated by applying the finite element analysis (FEA) technique to HRpQCT images.

While bone histomorphometry was not assessed in AD patients, data on BMD are controversial. Moreover, there is essentially no available information about bone structure in these patients. This is due, in part, to the fact that HRpQCT has not heretofore been applied to subjects with AD. This study aims to evaluate the bone health of patients with AD on chronic GC therapy using an HRpQCT technique. We hypothesized that such patients would have deteriorated bone microstructure and strength compared to controls.

## Materials and Methods

### Subjects

This cross-sectional study included postpubertal, nonpregnant individuals aged 16 years or older with an established diagnosis of primary adrenal insufficiency, defined by low basal serum cortisol levels (<5 mcg/dL) on at least 2 occasions and/or an impaired response to ACTH stimulation testing [6], in addition to classic clinical signs and symptoms. Participants were receiving GC replacement therapy for at least 1 year and were under routine follow-up at the Adrenal Clinic of the Federal University of Sao Paulo, Brazil.

Individuals with conditions or medications known to affect bone metabolism other than AD/GC were excluded. These included untreated hyperthyroidism, prolactinoma, Cushing's disease, primary hyperparathyroidism, hypoparathyroidism, osteomalacia, osteogenesis imperfecta, malabsorption syndromes, malignancies, chronic kidney or liver disease, organ transplantation, and conditions characterized by androgen excess (eg, congenital adrenal hyperplasia). Individuals with a history of bisphosphonate use (within the past 3 years), anticoagulants, methotrexate, aromatase inhibitors, thiazolidinediones, teriparatide, raloxifene, or denosumab were additionally excluded.

An age-, sex- and race-matched control group was included for comparison. The group was recruited through hospital flyers or by direct invitation. Controls were healthy individuals with no reported pathologies or use of medications that could significantly affect bone health as assessed by history. Race was defined by self-designation.

The study was approved by the Committee on Ethical Research of the Federal University of Sao Paulo. After being informed of the study's purposes and procedures, the participants signed a formal written consent form. Data were analyzed exclusively by the study researchers.

### Study Protocol

Data on previous fractures, use of medications (including GC, MC, and vitamin D supplements); calcium intake; and the duration, etiology, and treatment of AD were obtained from medical records and patient interviews. Different GCs were converted to hydrocortisone equivalents (mg/m²/day). The GC equivalence ratios were based on the doses that lead to suppression of the corticotrophic axis and growth, as follows: 80 mg of hydrocortisone = 16 mg of prednisone = 13.3 mg of prednisolone = 1 mg of dexamethasone [20, 21]. The cumulative GC dose was calculated since 2012, when electronic medical records were initiated at the institution. Anthropometric data, including weight and height, were recorded from all participants.

### Biochemical Analyses

Fasting morning blood samples were collected to measure biochemical parameters related to the adrenal and bone metabolism of AD patients. The laboratory evaluation was performed using standard methods for sodium, potassium, and total calcium. Plasma renin activity (PRA) was measured by ELISA, with intra- and interassay coefficients of variation of 4.1% and 11.0%, respectively. PTH and 25-hydroxyvitamin D were measured by electrochemiluminescence, as well as carboxyterminal telopeptide of type 1 collagen (CTX) and osteocalcin. Intra- and interassay coefficients of variation were, respectively, 3.0% and 3.5% for PTH, 8.5% and 9.2% for 25-hydroxyvitamin D, 1.1% and 1.2% for CTX, and 1.5% and 2.2% for osteocalcin (Elecsys—Cobas, Roche, Mannheim, Germany). Blood sample analyses were carried out in the same laboratory for all patients. All samples were stored at −20 °C.

### DXA Analyses

aBMD was measured at the lumbar spine (LS; L1-L4), total hip, femoral neck, and distal radius using DXA (Hologic, Waltham, MA, USA). Vertebral fracture assessment (VFA) and whole-body composition, including total body fat percentage (BF%), android/gynoid ratio, fat mass index (fat mass/height^2^), and appendicular skeletal muscle index (Baumgartner index = appendicular skeletal mass/height^2^) were also evaluated. While BF%, android/gynoid ratio, and fat mass index are fat indexes, the Baumgartner is a lean mass index (higher levels mean proportionally higher muscle mass) [22].

### HRpQCT Analyses

HRpQCT scans of the nondominant side were acquired using a second-generation system (XCT2, Scanco Medical, Brüttisellen, Switzerland). Limbs were secured in a manufacturer-provided fiber-carbon cast and positioned within the scanner's central cavity.

For each scan, a 2D x-ray image (scout view) was obtained to identify the region to be analyzed and to position the reference line (on the inflection point on the endplate of the distal radius and tibial plafond). Subsequently, using the standard manufacturer protocol, the XCT2 system acquired 168 slices of a 10.24 mm section with a 61 µm isotropic resolution, beginning 9.0 and 22.0 mm proximal to the reference line, respectively, at the radius and tibia [23]. The entire acquisition process took approximately 2 minutes per site.

Sectional and 3D images were generated. Cortical and trabecular bone compartments were segmented using a semiautomated technique. Subsequently, microarchitectural and volumetric density parameters were quantified for both compartments. Finite element analysis, through the parameter Stiffness, was used to assess mechanical properties.

### Statistical Analysis

The statistical analysis was performed using Microsoft Excel Version 16.87 (Microsoft, Redmond, WA, USA) and SAS Version 9.4 (SAS Institute, Cary, NC, USA). Absolute (n) and relative (percent) frequencies were measured for qualitative variables and mean ± SD or median (minimum-maximum) and 95% confidence intervals for quantitative variables.

Between-group comparisons using the chi-square test for categorical variables and a 2-sided *t*-test for continuous variables and standardized mean differences between groups were calculated. The significance level adopted was *P* ≤ .05. Adjustment for multiple comparisons for highly correlated trabecular HRpQCT parameters using Bonferroni correction was tested, as suggested in a recent publication [24]. Pearson correlations between clinical, biochemical, and bone parameters were performed in the AD group.

## Results

### Demographics, Clinical, Biochemical, and DXA Analyses

The descriptive and biochemical characteristics of the 19 AD patients are shown in Table 1. The median age was 52 years (24-82), 37% were women (71% postmenopausal), and 63% were White. The most common etiologies were autoimmune (63%) and infectious (16%). The median time since diagnosis was 6 years (2-33), and the initial age at GC treatment was 33 years (0-78). Six patients had a history of previous fractures, all in the extremities. No vertebral fractures were identified on imaging evaluation by VFA.

**Table 1.** Demographic, clinical, and biochemical characteristics of Addison’s disease patients, mean ± SD (n = 19)

		Reference values
Age (years)	50.8 ± 17.7	
Female, n (%)	7 (37)	
Race, n (%)		
White	12 (63)	
Black	3 (16)	
Brown	4 (21)	
Years since diagnosis	13.2 ± 11.2	
Addison's disease etiology, n (%)		
Autoimmune	12 (63)	
Infectious	3 (16)	
Other*^a^*	4 (21)	
Regular exercise, n (%)	10 (53)	
History of smoking cigarettes, n (%)	6 (32)	
History of alcohol abuse, n (%)	2 (11)	
Diabetes therapy with insulin, n (%)	3 (16)	
Previous fractures, n*^b^* (%)	6 (32)	
Prevalent vertebral fractures by vertebral fracture assessment, n	0	
Calcium intake (diet plus supplementation), mg/day	903 ± 440	
Vitamin D supplementation, n (%)	16 (84)	
Vitamin D supplementation dose IU/day	1675 ± 755	
Fludrocortisone use, n (%)	18 (95)	
Fludrocortisone dose, mcg/day	108 ± 65	
Age glucocorticoid was begun, years old	37.7 ± 19.6	
Current glucocorticoid, n (%)		
Prednisone	14 (74)	
Prednisolone	5 (26)	
Current glucocorticoid dose (hydrocortisone equivalence/body surface area), mg/m^2^/day	17.4 ± 6.0	
Cumulative glucocorticoid dose (hydrocortisone equivalence), grams	80.7 ± 50.3	
Sodium, mmol/L	138.6 ± 3.8	137-148
Potassium, mmol/L	5.0 ± 0.6	3.5-5.0
Plasma renin activity, ng/mL/h	6.1 ± 8.1	0.30-4.37
PTH, pg/mL	36.6 ± 11.3	15-65
Calcium, mg/dL	9.7 ± 0.5	8.6-10.2
Osteocalcin, ng/mL	18.0 ± 6.4	10-37
25OH-vitamin D, ng/mL	34.1 ± 11.3	20-60
s-CTX, ng/mL	0.305 ± 0.160	0.161-0.737

Abbreviations: CTX, carboxyterminal telopeptide of type 1 collagen.

^*a*^Other causes: 2 adrenoleukodystrophy, 1 idiopathic, 1 adrenal hypoplasia.

^*b*^Fracture locations: 1 wrist, 3 arm, 2 hand, 1 forearm.

Prednisone was used by 74% of patients, whereas the remaining took prednisolone. The mean daily GC dose was 29.6 ± 8.3 mg (17.4 ± 6.0 mg/m²), and the cumulative GC dose since 2012 was 80.7 ± 50.3 grams, expressed in hydrocortisone equivalents. All but 1 patient received fludrocortisone, with a mean daily dose of 108 ± 65 mcg. Sixteen patients (84%) took vitamin D supplements, and the estimated mean calcium intake (diet plus supplementation) was 903 ± 440 mg/day.

Despite MC (and GC) replacement, PRA was above the normal range, with potassium in the upper limit and sodium near the lower limit. All other blood parameters were within the normal range. The mean bone turnover markers (BTM) were within the reference range, with 4 patients presenting lower osteocalcin levels and 1 with lower CTX levels. No patient was above the reference range for BTM (Table 1).

The control group consisted of 38 individuals with no significant differences in anthropometric measurements compared to the AD group. Table 2 presents the group comparisons for DXA parameters. Compared to controls, patients with AD had lower areal BMD at the LS, femoral neck, and total hip by 10.3%, 10.8%, and 9.5%, respectively, with no difference at the radius.

**Table 2.** Anthropometric and regional BMD and body composition by DXA in Addison’s patients and healthy controls, mean ± SD (95% CI)

	Addison’s disease, n = 19	Control, n = 38	*P*-value (SMD)
Age, years	50.8 ± 17.7	48.3 ± 14.7	.57
Female, n (%)	7 (37)	14 (37)	1.00
Race, n (%)			
White	12 (63)	24 (63)	.83
Black	3 (16)	8 (21)	
Brown	4 (21)	6 (16)	
Height, m	1.63 ± 0.11	1.65 ± 0.10	.42
Weight, kg	68.9 ± 13.2	71.9 ± 12.9	.42
BMI, kg/m^2^	26.0 ± 4.5	26.3 ± 4.4	.80
Lumbar spine, g/cm^2^	0.942 ± 0.131 (0.879, 1.005)	1.051 ± 0.17 (0.996, 1.107)	**.02 (−0.69)**
T-score	−1.1 ± 1.2 (−1.7, −0.6)	−0.2 ± 1.5 (−0.7, 0.3)	**.02 (−0.66)**
Z-score	−0.3 ± 1.3 (−0.9, 0.4)	0.3 ± 1.7 (−0.2, 0.9)	.19 (−0.37)
Femoral neck, g/cm^2^	0.753 ± 0.132 (0.690, 0.817)	0.845 ± 0.157 (0.794, 0.897)	**.03 (−0.62)**
T-score	−1.1 ± 1.0 (−1.6, −0.7)	−0.4 ± 1.2 (−0.8, −0.1)	**.03 (−0.63)**
Z-score	−0.3 ± 1.0 (−0.8, 0.2)	0.3 ± 1.2 (−0.1, 0.6)	.10 (−0.47)
Total hip, g/cm^2^	0.857 ± 0.144 (0.787, 0.927)	0.947 ± 0.145 (0.899, 0.995)	**.03 (−0.62)**
T-score	−1.0 ± 0.9 (−1.5, −0.6)	−0.4 ± 1.0 (−0.7, 0.0)	**.02 (−0.66)**
Z-score	−0.4 ± 0.9 (−0.9, 0.0)	0.1 ± 1.0 (−0.3, 0.4)	.07 (−0.51)
Distal radius, g/cm^2^	0.709 ± 0.180 (0.622, 0.796)	0.716 ± 0.082 (0.689, 0.743)	.88 (−0.06)
T-score	−1.4 ± 1.6 (−2.2, −0.6)	−1.0 ± 1.1 (−1.4, −0.6)	.32 (−0.28)
Z-score	−0.5 ± 1.7 (−1.3, 0.3)	−0.4 ± 1.1 (−0.7, 0.0)	.76 (−0.10)
Body fat, %	35.6 ± 12.7 (29.5, 41.7)	30.0 ± 9.0 (27.0, 32.9)	.07 (0.54)
Android to gynoid ratio	1.00 ± 0.14 (0.93, 1.07)	1.09 ± 0.28 (1.00, 1.18)	.13 (−0.36)
Fat mass index, kg/m^2^	9.4 ± 4.7 (7.1, 11.7)	7.9 ± 3.3 (6.8, 9.0)	.19 (0.38)
Baumgartner, kg/m^2^	6.39 ± 1.09 (5.86, 6.91)	7.39 ± 1.32 (6.95, 7.82)	**<.01 (−0.80)**

Bold numbers indicate *P* ≤ .05.

Abbreviations: BMD, bone mineral density; BMI, body mass index; CI, confidence interval; DXA, dual-energy x-ray absorptiometry; SMD, standardized mean difference.

The Baumgartner index was 13.5% lower in the AD patients (*P* < .01) with no differences in the fat indexes (Table 2), although there was a trend to higher BF% in the AD group (35.6 ± 12.7 vs 30.0 ± 9.0, *P* = .07).

### HRpQCT Comparisons

As shown in Table 3, there were several between-group differences in bone microarchitectural parameters, more significant at the tibia. At this site, AD patients demonstrated lower total volumetric BMD, trabecular vBMD (TbvBMD), trabecular bone volume fraction (TbBV/TV), and trabecular number (TbN), with higher trabecular separation (TbSp) (18-26% difference in these parameters, *P* < .02 for all). There was also a trend toward decreased trabecular thickness (TbTh). Cortical differences at the tibia were also observed, with 22% and 14% lower cortical thickness and cortical area, respectively, and a trend toward decreased cortical vBMD. Correcting for multiple comparisons between highly correlated trabecular parameters (TbBV/TV, TbN, TbTh, TbSp) using the Bonferroni correction with a *P*-value threshold of .0125, the differences remained significant.

**Table 3.** HRpQCT-derived bone parameters at the distal radius and tibia in Addison’s disease patients and controls, mean ± SD (95% CI)

Bone parameter	Addison’s disease, n = 19	Control, n = 38	*P*-value (SMD)
Distal radius parameters			
Cortical porosity, %	0.67 ± 0.38 (0.49, 0.85)	0.77 ± 0.66 (0.55, 0.99)	.47 (−0.17)
Cortical volumetric BMD, mgHA/cm^3^	897.0 ± 57.2 (869.4, 924.6)	916.8 ± 59.1 (897.3, 936.2)	.23 (−0.34)
Cortical thickness, mm	1.07 ± 0.30 (0.93, 1.22)	1.16 ± 0.23 (1.08, 1.24)	.24 (−0.33)
Cortical area, mm^2^	63.2 ± 20.8 (53.2, 73.3)	69.0 ± 14.6 (64.3, 73.8)	.23 (−0.34)
Trabecular bone volume fraction	0.221 ± 0.075 (0.185, 0.257)	0.260 ± 0.073 (0.236, 0.284)	.07 (−0.53)
Trabecular number, 1/mm	1.35 ± 0.25 (1.23, 1.47)	1.50 ± 0.23 (1.42, 1.57)	**.03** (**−0.62)**
Trabecular thickness, mm	0.23 ± 0.03 (0.22, 0.25)	0.24 ± 0.02 (0.23, 0.24)	.53 (−0.18)
Trabecular separation, mm	0.73 ± 0.21 (0.63, 0.83)	0.62 ± 0.12 (0.58, 0.67)	.053 (0.67)
Trabecular volumetric BMD, mgHA/cm^3^	152.5 ± 51.9 (127.5, 177.5)	180.3 ± 48.9 (164.3, 196.4)	.052 (−0.56)
Trabecular area, mm^2^	215.1 ± 71.6 (180.6, 249.6)	218.6 ± 61.0 (198.5, 238.6)	.85 (−0.05)
Total area, mm^2^	274.6 ± 76.2 (237.9, 311.3)	283.8 ± 64.6 (262.6, 305.1)	.63 (−0.13)
Stiffness, kN/mm	70.2 ± 30.0 (55.7, 84.6)	81.0 ± 25.8 (72.5, 89.4)	.16 (−0.40)
Distal tibia parameters			
Cortical porosity, %	2.71 ± 1.29 (2.08, 3.33)	2.40 ± 1.66 (1.85, 2.95)	.49 (0.20)
Cortical volumetric BMD, mgHA/cm^3^	879.5 ± 60.9 (850.1, 908.8)	911.7 ± 60.1 (891.9, 931.4)	.06 (−0.53)
Cortical thickness, mm	1.35 ± 0.32 (1.19, 1.50)	1.64 ± 0.34 (1.53, 1.76)	**.003** (**−0.89)**
Cortical area, mm^2^	121.7 ± 32.2 (106.2, 137.3)	141.9 ± 29.5 (132.2, 151.6)	**.02** (**−0.66)**
Trabecular bone volume fraction	0.217 ± 0.047 (0.194, 0.240)	0.265 ± 0.063 (0.244, 0.286)	**.005** (**−0.82)**
Trabecular number, 1/mm	1.11 ± 0.20 (1.01, 1.20)	1.30 ± 0.24 (1.22, 1.38)	**.004** (**−0.85)**
Trabecular thickness, mm	0.25 ± 0.02 (0.24, 0.26)	0.27 ± 0.02 (0.26, 0.27)	.055 (−0.55)
Trabecular separation, mm	0.91 ± 0.24 (0.80, 1.03)	0.75 ± 0.15 (0.70, 0.80)	**.01** (**0.88)**
Trabecular volumetric BMD, mgHA/cm^3^	142.6 ± 35.1 (125.7, 159.4)	179.3 ± 46.1 (164.1, 194.4)	**.003** (**−0.86)**
Trabecular area, mm^2^	617.6 ± 130.2 (554.8, 680.3)	554.3 ± 139.5 (508.4, 600.1)	.10 (0.46)
Total area, mm^2^	733.8 ± 137.6 (667.5, 800.1)	690.9 ± 149.2 (641.9, 740.0)	.30 (0.29)
Stiffness, kN/mm	177.5 ± 57.1 (150.0, 205.0)	225.1 ± 103.6 (191.0, 259.1)	**.03** (**−0.52)**

Bold numbers indicate *P* ≤ .05.

Abbreviations: CI, confidence interval; HRpQCT, high-resolution peripheral quantitative computed tomography; SMD, standardized mean difference.

No significant differences were observed between groups in cortical parameters at the radius, although the trabecular compartment appeared slightly more affected in the AD group, with 11% lower TbN and a trend toward reduced TbvBMD and TbBV/TV, together with increased TbSp.

**Figure 1.**
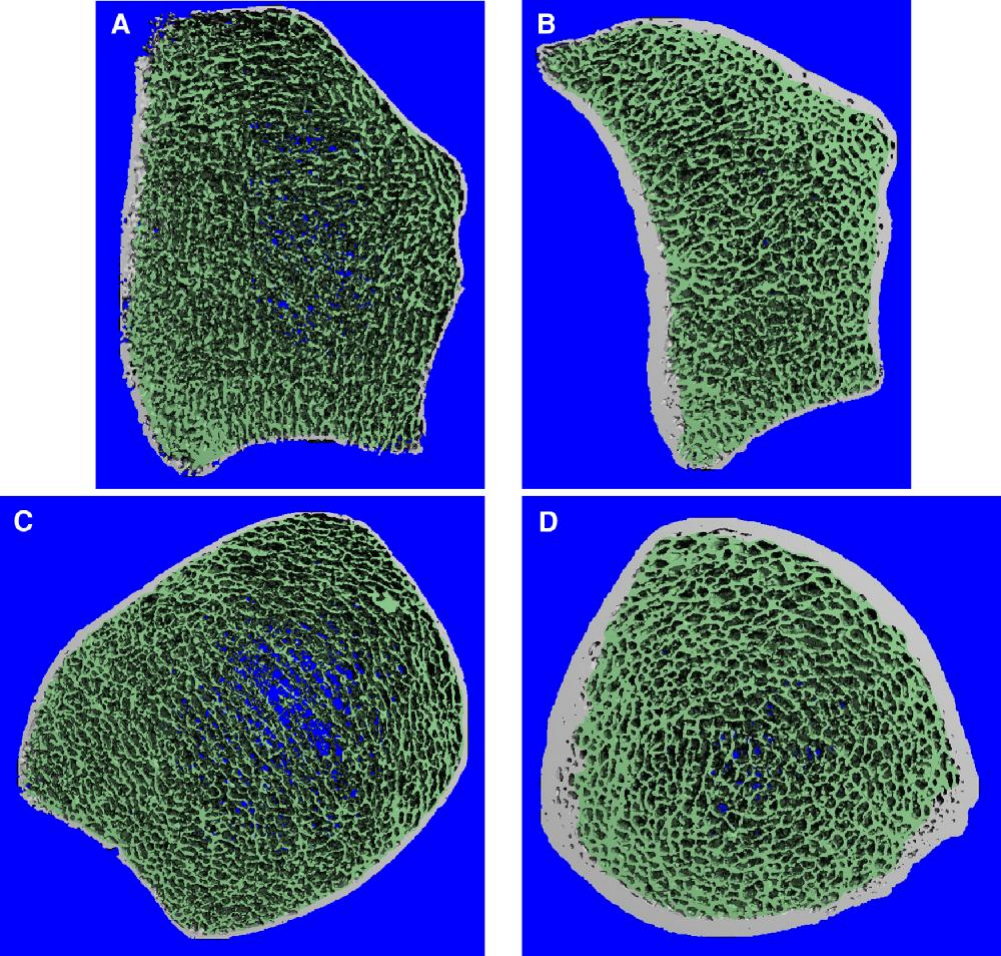
HRpQCT images of the radius (A) and tibia (C) of a 24-year-old Addison patient, and the radius (B) and tibia (D) of a 21-year-old healthy control, both males.

FEA revealed 21.1% lower tibia stiffness in the AD group (*P* < .03), with no significant difference at the radius. Figure 1 illustrates HRpQCT scans of 1 AD patient and 1 healthy control, both males of similar age.

### Correlations Concerning Bone Health

**Figure 2.**
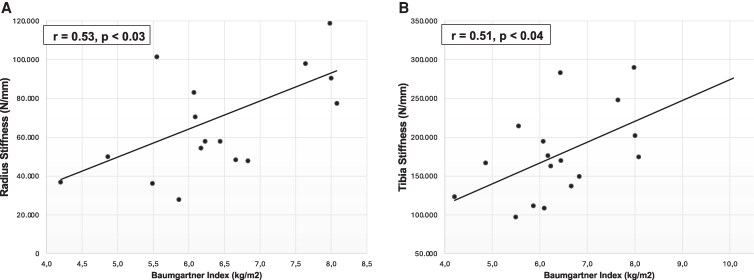
Scatter plots showing positive correlations between the Baumgartner Index and stiffness at both the radius (A) and tibia (B) in Addison’s disease patients.

In the AD group, we found a moderate positive correlation between the Baumgartner index and stiffness at both the radius and tibia, indicating that greater muscle mass was associated with better mechanical competence (Fig. 2). Several microarchitectural parameters, including both trabecular and cortical, showed moderate negative correlations with sodium levels and positive correlations with potassium and PRA levels (Table 4). There was no significant correlation between fludrocortisone or BTM and any HRpQCT parameter.

**Table 4.** Correlations between Na, K, PRA, and HRpQCT parameters in the Addison’s disease group

	Radius TotvBMD		Radius TbvBMD		Radius TbTh		Radius CtAr		Radius CtTh		Tibia TotvBMD		Tibia CtTh	
	r	*P*	r	*P*	r	*P*	r	*P*	r	*P*	r	*P*	r	*P*
Na	−0.66	.**005**	−0.51	.**04**	−0.71	.**002**	−0.53	.**04**	−0.66	.**006**	−0.64	.**007**	−0.68	.**004**
K	0.56	.**03**	0.54	.**04**	0.60	.**02**	0.55	.**03**	0.58	.**02**	0.41	.13	0.49	.06
PRA	0.72	.**001**	0.55	.**03**	0.62	.**01**	0.51	.**04**	0.67	.**004**	0.62	.**01**	0.52	.**04**

Bold numbers indicate *P* ≤ .05.

Abbreviations: CtAr, cortical area; CtTh, cortical thickness; HRpQCT, high-resolution peripheral quantitative computed tomography; K, potassium; Na, sodium; PRA, plasma renin activity; TbTh, trabecular thickness; TbvBMD, trabecular volumetric bone mineral density; TotvBMD, total volumetric bone mineral density.

**Figure 3.**
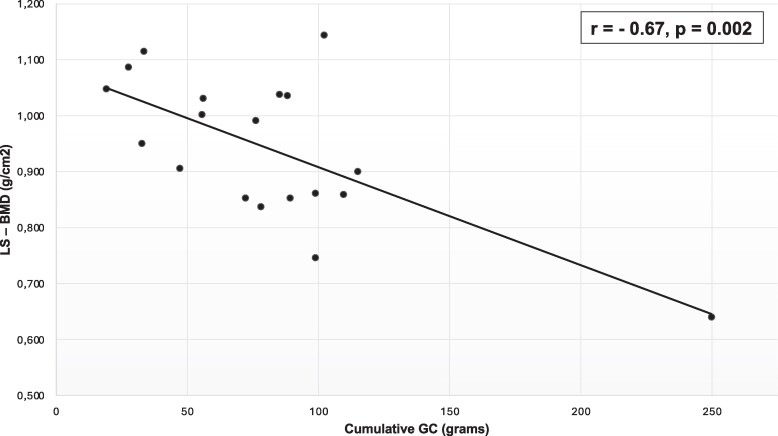
Scatter plot showing a negative correlation between cumulative glucocorticoid and lumbar spine bone mineral density in Addison’s disease patients.

There were no significant correlations between bone microarchitecture parameters and disease duration, although we observed some trends in tibia total vBMD (r = −0.45, *P* = .054) and tibia cortical thickness (r = −0.43, *P* = .067). However, cumulative GC dose was moderately negatively correlated with spine aBMD (r = −0.67, *P* < .01), as illustrated in Fig. 3. This correlation remained statistically significant after excluding an outlier patient (r = −0.45, *P* < .03).

## Discussion

To our knowledge, this is the first study to assess bone microarchitecture using HRpQCT in patients with AD. Our results indicate that AD patients on GC have reduced aBMD, impaired bone microarchitecture, and lower muscle mass, which was more pronounced in the trabecular compartment and at the tibia. This was associated with reduced strength at the tibia. These results imply that patients with AD on GC replacement are at risk for the consequence of more fragile bone.

Previous studies are conflicting with regard to aBMD in patients with AD. Some show no difference [25-27], while others indicate lower aBMD, especially in postmenopausal women [28, 29], hypogonadal men [30, 31], and those on more potent GC (prednisolone compared to hydrocortisone) [32] or higher GC doses [33]. In our study, AD patients had lower aBMD at the LS and hip sites compared to controls. This could be anticipated, as none of our patients were on low-potency steroids (hydrocortisone), and most women were postmenopausal. In addition, AD patients used a mean hydrocortisone equivalent GC dose of 17.4 mg/m²/day, which is above physiologic levels, estimated as 9 to 11 mg/m²/day [34-36]. Some studies suggest that aBMD impairment occurs when the GC dose is above 12 to 13 mg/m²/day in AD patients on long-term treatment [33, 37].

Bone mineral density at the LS was inversely correlated with cumulative GC dose, a finding previously observed [27, 37, 38] that corroborates the relationship between GC and bone loss in AD patients.

We also found that AD patients have impaired microarchitectural parameters. Bone microarchitecture in AD patients has only been evaluated previously with trabecular bone score (TBS) [39]. A study comparing AD patients with matched controls showed no differences in TBS (1.32 ± 0.1 vs 1.30 ± 0.1, *P* = .63). Unlike ours, the study showed no difference in aBMD between the groups [27]. Compared to our data, the patients in the study had similar age and disease duration. However, the mean GC dose (25.8 ± 6.2 mg/day) was lower than that of our patients (29.6 ± 8.3 mg/day), which may explain the neutral aBMD results. Nevertheless, the analysis showed an inverse correlation between TBS and disease duration in AD patients (r = −0.48, *P* = .009), suggesting that prolonged GC use may be related to microarchitecture deterioration [27]. TBS, as an indirect method, has lower sensitivity to detect microarchitectural changes than HRpQCT and is just weakly to moderately associated with microarchitectural and FEA indices by HRpQCT [40-42].

Data from other conditions that require chronic GC use have shown impaired microarchitecture by TBS [43, 44] and HRpQCT [45-47], although it's important to highlight that these patients use GC at anti-inflammatory, not replacement, doses.

Notwithstanding, patients with endogenous hypercortisolism, even subclinical, also seem to have microarchitectural skeletal impairment. In a study with 75 patients with adrenal incidentalomas, those with subclinical hypercortisolism, compared to those with nonfunctioning nodules, had lower aBMD and worse trabecular architecture (lower TbvBMD, TbBV/TV, and TbTh) at the radius but not at the tibia [48], as assessed by HRpQCT. These data suggest that even minimal increases in cortisol levels may be associated with microstructural deterioration, endorsing our results.

Microarchitectural abnormalities and decreased BMD were accompanied by impaired bone strength by FEA at the tibia in our patients. Data with chronic GC users on anti-inflammatory doses have also demonstrated decreased stiffness [45, 46]. The reduction of bone strength observed in our study suggests that GC-treated AD patients are at increased risk for fracture [49, 50]. Studies that have assessed fracture risk in AD are consistent with this finding. Bjornsdottir et al found a higher risk of hip fractures in AD patients compared to controls (hazard ratio = 1.8, 95% confidence interval 1.6-2.1, *P* < .001), especially in females diagnosed longer [51]. Camozzi et al demonstrated that AD patients have more than a 3-fold risk of vertebral fractures as compared to healthy individuals, and the risk was also associated with a longer disease duration [52]. Although our patients had lower bone stiffness than controls, none had a vertebral fracture by VFA or a history of hip fractures. Nonetheless, 32% had a history of limb fractures.

Consistent with prior studies, mean BTM levels were within the reference range in our patients [25, 29, 37, 38]. In contrast, in individuals using supraphysiologic anti-inflammatory doses of GC, a decrease in formation markers is observed [53-55], consistent with the long-term effect of GCs to reduce bone formation. These differences may be due, at least in part, to differences in the mean GC dose. Most AD patients use replacement GC doses, which are often above the physiologic level but not high enough to suppress formation markers. Yet, a few patients in our study had suppressed BTM and mean osteocalcin levels that were in the lower normal range. The normal levels of PTH and CTX suggest no remarkable increases in bone resorption or urinary calcium loss.

Changes in body composition in patients using supraphysiologic GC doses are well recognized [56-58]. However, it is notable that even replacement doses of GC can lead to body composition changes, as our patients had remarkably lower muscle mass and a trend toward higher BF% compared to healthy individuals.

A relationship between muscle mass/strength and aBMD has been previously observed in anti-inflammatory doses GC users [56, 58] and non-GC users [59]. In older [60] and obese [61] adults, the lean mass explained 28% to 48% of variance in bone strength. However, the relationship between muscle mass and bone microstructure or strength in individuals on GC has not been investigated so far. We demonstrated a positive correlation between muscle mass and bone stiffness in AD patients on replacement GC.

Decreased muscle mass in patients with AD may be due, in part, to GC use, a condition described as “steroid myopathy” [62]. Our data suggest that patients with AD, on “replacement doses“of GC that do not affect BTM, usually use supraphysiological doses and are not exempt from musculoskeletal impairment caused by the GC. In addition, adrenal insufficiency may be associated with higher levels of myostatin, a protein that negatively regulates muscle mass, as suggested in a preclinical study [63]. The fact that the mean BTMs are normal, with significant muscle loss (and a correlation between lean mass and bone stiffness), may suggest that these body composition changes play an important role in the pathophysiology of bone impairment of AD patients on replacement GC.

The degradation of the microarchitecture was surprisingly more prominent at the tibia. One might expect that bone damage could be mitigated at this site, since it is a weight-bearing area, as observed in studies with endogenous hypercortisolism and general GC users [45, 48]. Further studies are needed to confirm and possibly explain this difference. We hypothesize that the loss of lean mass would affect predominantly the lower limb skeleton, as it usually receives more muscle stimulus and mechanical stress than the upper limbs.

One possible confounding effect for our results is the gonadal status, especially in men, as they comprised most of our patients. In addition to directly affecting bone health, chronic GC use may decrease testosterone levels, which can potentiate bone degradation [12, 64]. Studies assessing aBMD in AD patients show that hypogonadal men are among the populations at risk for bone loss [30, 31]. Furthermore, the drop in testosterone levels intensifies muscle loss, which also contributes to bone impairment [12, 64]. As we did not measure testosterone levels, we could not determine the gonadal status of the male participants.

Microarchitectural parameters correlated negatively with sodium and positively with potassium and PRA. We postulate that these correlations are due to the GC action on mineralocorticoid receptors, so the higher the GC dose, the higher the sodium and the lower the potassium and PRA, and the worse the bone parameters. Also, mineralocorticoids have a potential impact on bone, leading to urinary calcium loss (calcium/sodium transport is coupled in the nephron), as observed in patients with primary aldosteronism [65, 66]. Although 95% of our patients were on fludrocortisone, they were not on excessive doses (as verified by normal potassium and not suppressed PRA); it may, however, have contributed to bone impairment.

Our study has several strengths. It is the first study to assess bone microstructure using HRpQCT in patients with AD. We used the second-generation HRpQCT instrument, which has superior image resolution compared to first-generation scanners and provides a direct measurement of trabecular thickness and separation (these are measured indirectly on XCT1). Moreover, the XCT2 scan is acquired faster, resulting in fewer movement artifacts [17, 18]. In addition to volumetric BMD and microarchitecture, we assessed bone strength using FEA. Although we used the standard acquisition protocol and not a relative offset, our cases and controls were height-matched to ensure the region of interest was the same.

Our study also has some limitations. It is a cross-sectional study with a relatively small, convenience-based sample size. As this is the first study using HRpQCT in AD, we did not perform a power calculation for sample size. However, the current sample size is similar to many other HRpQCT studies, and we could detect several differences in bone parameters. Due to the relatively small sample size, we did not analyze the data separately for men and women. However, patients and controls were sex matched. Another limitation is the lack of control group biochemical data for comparison, although this group was healthy, and there were no remarkable changes in the biochemical parameters of the patients. Finally, the cumulative GC dose may have been underestimated since the information was only available from 2012. Even so, we found an inverse correlation with aBMD.

In conclusion, this is the first study that presents data on bone microarchitecture, as measured by HRpQCT, in patients with AD. Beyond the bone and lean mass loss by DXA, our findings suggest that AD patients on chronic GC replacement therapy have bone microarchitectural and strength abnormalities, more pronounced in the trabecular bone and at the distal tibia. Bone fragility may be related to muscle loss and cumulative GC dose. Our data highlight the importance of assessing bone health in patients with AD and suggest clinicians should address and prevent muscle loss, avoiding high GC doses as much as possible.

## Data Availability

Some or all datasets generated during and/or analyzed during the current study are not publicly available but are available from the corresponding author on reasonable request.

## References

[1] Younes N, Bourdeau I, Lacroix A. Latent adrenal insufficiency: from concept to diagnosis. Front Endocrinol (Lausanne). 2021;12:720769. 34512551 10.3389/fendo.2021.720769 PMC8429826

[2] Oelkers W . Adrenal insufficiency. N Engl J Med. 1996;335(16):1206‐1212. 8815944 10.1056/NEJM199610173351607

[3] Bancos I, Hahner S, Tomlinson J, Arlt W. Diagnosis and management of adrenal insufficiency. Lancet Diabetes Endocrinol. 2015;3(3):216‐226. 25098712 10.1016/S2213-8587(14)70142-1

[4] Silva Rdo C, Castro M, Kater CE, et al [Primary adrenal insufficiency in adults: 150 years after Addison]. Arq Bras Endocrinol Metabol. 2004;48(5):724‐738. 15761544 10.1590/s0004-27302004000500019

[5] Agarwal G, Bhatia E, Pandey R, Jain SK. Clinical profile and prognosis of Addison's disease in India. Natl Med J India. 2001;14(1):23‐25. 11242694

[6] Bornstein SR, Allolio B, Arlt W, et al Diagnosis and treatment of primary adrenal insufficiency: an endocrine society clinical practice guideline. J Clin Endocrinol Metab. 2016;101(2):364‐389. 26760044 10.1210/jc.2015-1710 PMC4880116

[7] Debono M, Ross RJ, Newell-Price J. Inadequacies of glucocorticoid replacement and improvements by physiological circadian therapy. Eur J Endocrinol. 2009;160(5):719‐729. 19168600 10.1530/EJE-08-0874

[8] Løvås K, Loge JH, Husebye ES. Subjective health status in Norwegian patients with Addison's disease. Clin Endocrinol (Oxf). 2002;56(5):581‐588. 12030907 10.1046/j.1365-2265.2002.01466.x

[9] Skov J, Sundström A, Ludvigsson JF, Kämpe O, Bensing S. Sex-specific risk of cardiovascular disease in autoimmune addison disease-A population-based cohort study. J Clin Endocrinol Metab. 2019;104(6):2031‐2040. 30608542 10.1210/jc.2018-02298 PMC6469226

[10] Compston J . Glucocorticoid-induced osteoporosis: an update. Endocrine. 2018;61(1):7‐16. 29691807 10.1007/s12020-018-1588-2 PMC5997116

[11] Dalle Carbonare L, Chavassieux PM, Arlot ME, Meunier PJ. Bone histomorphometry in untreated and treated glucocorticoid-induced osteoporosis. Front Horm Res. 2002;30:37‐48. 11892269 10.1159/000061069

[12] Shaker JL, Lukert BP. Osteoporosis associated with excess glucocorticoids. Endocrinol Metab Clin North Am. 2005;34(2):341‐356. 15850846 10.1016/j.ecl.2005.01.014

[13] Raisz LG, Kream BE. Regulation of bone formation. N Engl J Med. 1983;309(1):29‐35. 6343872 10.1056/NEJM198307073090107

[14] Hahn TJ, Halstead LR, Baran DT. Effects off short term glucocorticoid administration on intestinal calcium absorption and circulating vitamin D metabolite concentrations in man. J Clin Endocrinol Metab. 1981;52(1):111‐115. 6969728 10.1210/jcem-52-1-111

[15] Eastell R, Rosen CJ, Black DM, Cheung AM, Murad MH, Shoback D. Pharmacological management of osteoporosis in postmenopausal women: an endocrine society* clinical practice guideline. J Clin Endocrinol Metab. 2019;104(5):1595‐1622. 30907953 10.1210/jc.2019-00221

[16] Black DM, Cummings SR, Genant HK, Nevitt MC, Palermo L, Browner W. Axial and appendicular bone density predict fractures in older women. J Bone Miner Res. 1992;7(6):633‐638. 1414481 10.1002/jbmr.5650070607

[17] Fuller H, Fuller R, Pereira RM. [High resolution peripheral quantitative computed tomography for the assessment of morphological and mechanical bone parameters]. Rev Bras Reumatol. 2015;55(4):352‐362. 25582999 10.1016/j.rbr.2014.07.010

[18] Cheung AM, Adachi JD, Hanley DA, et al High-resolution peripheral quantitative computed tomography for the assessment of bone strength and structure: a review by the Canadian bone strength working group. Curr Osteoporos Rep. 2013;11(2):136‐146. 23525967 10.1007/s11914-013-0140-9 PMC3641288

[19] Lewis MK, Blake GM, Fogelman I. Patient dose in dual x-ray absorptiometry. Osteoporos Int. 1994;4(1):11‐15. 8148566 10.1007/BF02352255

[20] Caldato MC, Fernandes VT, Kater CE. One-year clinical evaluation of single morning dose prednisolone therapy for 21-hydroxylase deficiency. Arq Bras Endocrinol Metabol. 2004;48(5):705‐712. 15761542 10.1590/s0004-27302004000500017

[21] Rivkees SA . Dexamethasone therapy of congenital adrenal hyperplasia and the myth of the “growth toxic” glucocorticoid. Int J Pediatr Endocrinol. 2010;2010:569680. 20414340 10.1155/2010/569680 PMC2855951

[22] Maeda SS, Albergaria BH, Szejnfeld VL, et al Official position of the Brazilian Association of Bone Assessment and Metabolism (ABRASSO) on the evaluation of body composition by densitometry-part II (clinical aspects): interpretation, reporting, and special situations. Adv Rheumatol. 2022;62(1):11. 35365246 10.1186/s42358-022-00240-9

[23] Whittier DE, Boyd SK, Burghardt AJ, et al Guidelines for the assessment of bone density and microarchitecture in vivo using high-resolution peripheral quantitative computed tomography. Osteoporos Int. 2020;31(9):1607‐1627. 32458029 10.1007/s00198-020-05438-5 PMC7429313

[24] Neeteson NJ, Bugbird AR, Burt LA, Boyd SK. Statistical investigation of interdependence of trabecular microarchitectural parameters from micro-computed tomography. Bone. 2024;187:117144. 38834103 10.1016/j.bone.2024.117144

[25] Jódar E, Valdepeñas MP, Martinez G, Jara A, Hawkins F. Long-term follow-up of bone mineral density in Addison's disease. Clin Endocrinol (Oxf). 2003;58(5):617‐620. 12699444 10.1046/j.1365-2265.2003.01761.x

[26] Arlt W, Rosenthal C, Hahner S, Allolio B. Quality of glucocorticoid replacement in adrenal insufficiency: clinical assessment vs. timed serum cortisol measurements. Clin Endocrinol (Oxf). 2006;64(4):384‐389. 16584509 10.1111/j.1365-2265.2006.02473.x

[27] Zdrojowy-Wełna A, Halupczok-Żyła J, Słoka N, Syrycka J, Gojny Ł, Bolanowski M. Trabecular bone score and sclerostin concentrations in patients with primary adrenal insufficiency. Front Endocrinol (Lausanne). 2022;13:996157. 36407318 10.3389/fendo.2022.996157 PMC9666397

[28] Devogelaer JP, Crabbé J, de Deuxchaisnes CN. Bone mineral density in Addison's disease: evidence for an effect of adrenal androgens on bone mass. Br Med J (Clin Res Ed). 1987;294(6575):798‐800. 10.1136/bmj.294.6575.798 PMC1245862 2952217

[29] Valero MA, Leon M, Ruiz Valdepeñas MP, et al Bone density and turnover in Addison's disease: effect of glucocorticoid treatment. Bone Miner. 1994;26(1):9‐17. 7950508 10.1016/s0169-6009(08)80158-4

[30] Braatvedt GD, Joyce M, Evans M, Clearwater J, Reid IR. Bone mineral density in patients with treated Addison's disease. Osteoporos Int. 1999;10(6):435‐440. 10663342 10.1007/s001980050251

[31] Chandy DD, Bhatia E. Bone mineral density in patients with addison disease on replacement therapy with prednisolone. Endocr Pract. 2016;22(4):434‐439. 26684152 10.4158/EP151014.OR

[32] Koetz KR, Ventz M, Diederich S, Quinkler M. Bone mineral density is not significantly reduced in adult patients on low-dose glucocorticoid replacement therapy. J Clin Endocrinol Metab. 2012;97(1):85‐92. 21994966 10.1210/jc.2011-2036

[33] Chikada N, Imaki T, Hotta M, Sato K, Takano K. An assessment of bone mineral density in patients with Addison's disease and isolated ACTH deficiency treated with glucocorticoid. Endocr J. 2004;51(3):355‐360. 15256782 10.1507/endocrj.51.355

[34] Linder BL, Esteban NV, Yergey AL, Winterer JC, Loriaux DL, Cassorla F. Cortisol production rate in childhood and adolescence. J Pediatr. 1990;117(6):892‐896. 2104527 10.1016/s0022-3476(05)80128-3

[35] Esteban NV, Yergey AL. Cortisol production rates measured by liquid chromatography/mass spectrometry. Steroids. 1990;55(4):152‐158. 2187284 10.1016/0039-128x(90)90103-i

[36] Kerrigan JR, Veldhuis JD, Leyo SA, Iranmanesh A, Rogol AD. Estimation of daily cortisol production and clearance rates in normal pubertal males by deconvolution analysis. J Clin Endocrinol Metab. 1993;76(6):1505‐1510. 8501158 10.1210/jcem.76.6.8501158

[37] Yazidi M, Danguir C, Maamer D, et al Impact of hydrocortisone replacement on bone mineral density and bone turnover markers in patients with primary adrenal insufficiency. Endocr Regul. 2022;56(3):209‐215. 35843715 10.2478/enr-2022-0022

[38] Furman K, Gut P, Sowińska A, Ruchała M, Fichna M. Predictors of bone mineral density in patients receiving glucocorticoid replacement for Addison's disease. Endocrine. 2024;84(2):711‐719. 38334892 10.1007/s12020-024-03709-3

[39] Silva BC, Leslie WD, Resch H, et al Trabecular bone score: a noninvasive analytical method based upon the DXA image. J Bone Miner Res. 2014;29(3):518‐530. 24443324 10.1002/jbmr.2176

[40] Silva BC, Walker MD, Abraham A, et al Trabecular bone score is associated with volumetric bone density and microarchitecture as assessed by central QCT and HRpQCT in Chinese American and white women. J Clin Densitom. 2013;16(4):554‐561. 24080513 10.1016/j.jocd.2013.07.001 PMC3818347

[41] Alvarenga JC, Boyd SK, Pereira RMR. The relationship between estimated bone strength by finite element analysis at the peripheral skeleton to areal BMD and trabecular bone score at lumbar spine. Bone. 2018;117:47‐53. 30219479 10.1016/j.bone.2018.09.009

[42] Popp AW, Buffat H, Eberli U, et al Microstructural parameters of bone evaluated using HR-pQCT correlate with the DXA-derived cortical index and the trabecular bone score in a cohort of randomly selected premenopausal women. PLoS One. 2014;9(2):e88946. 24551194 10.1371/journal.pone.0088946 PMC3923873

[43] Lee KA, Kim J, Kim HJ, Kim HS. Discriminative ability of trabecular bone score over bone mineral density for vertebral and fragility fracture in patients treated with long-term and low-dose glucocorticoid. Int J Rheum Dis. 2021;24(8):1053‐1060. 34184827 10.1111/1756-185X.14164

[44] Paggiosi MA, Peel NF, Eastell R. The impact of glucocorticoid therapy on trabecular bone score in older women. Osteoporos Int. 2015;26(6):1773‐1780. 25743176 10.1007/s00198-015-3078-1

[45] Sutter S, Nishiyama KK, Kepley A, et al Abnormalities in cortical bone, trabecular plates, and stiffness in postmenopausal women treated with glucocorticoids. J Clin Endocrinol Metab. 2014;99(11):4231‐4240. 25127089 10.1210/jc.2014-2177 PMC4223438

[46] Tang XL, Qin L, Kwok AW, et al Alterations of bone geometry, density, microarchitecture, and biomechanical properties in systemic lupus erythematosus on long-term glucocorticoid: a case-control study using HR-pQCT. Osteoporos Int. 2013;24(6):1817‐1826. 23104200 10.1007/s00198-012-2177-5

[47] Jin S, Li M, Wang Q, et al Bone mineral density and microarchitecture among Chinese patients with rheumatoid arthritis: a cross-sectional study with HRpQCT. Arthritis Res Ther. 2021;23(1):127. 33894786 10.1186/s13075-021-02503-0 PMC8067377

[48] Moraes AB, de Paula MP, de Paula Paranhos-Neto F, et al Bone evaluation by high-resolution peripheral quantitative computed tomography in patients with adrenal incidentaloma. J Clin Endocrinol Metab. 2020;105(8):e2726‐e2737. 10.1210/clinem/dgaa263 32413110

[49] Vilayphiou N, Boutroy S, Sornay-Rendu E, et al Finite element analysis performed on radius and tibia HR-pQCT images and fragility fractures at all sites in postmenopausal women. Bone. 2010;46(4):1030‐1037. 20044044 10.1016/j.bone.2009.12.015

[50] Vilayphiou N, Boutroy S, Szulc P, et al Finite element analysis performed on radius and tibia HR-pQCT images and fragility fractures at all sites in men. J Bone Miner Res. 2011;26(5):965‐973. 21541999 10.1002/jbmr.297

[51] Björnsdottir S, Sääf M, Bensing S, Kämpe O, Michaëlsson K, Ludvigsson JF. Risk of hip fracture in Addison's disease: a population-based cohort study. J Intern Med. 2011;270(2):187‐195. 21251095 10.1111/j.1365-2796.2011.02352.x

[52] Camozzi V, Betterle C, Frigo AC, et al Vertebral fractures assessed with dual-energy X-ray absorptiometry in patients with Addison's disease on glucocorticoid and mineralocorticoid replacement therapy. Endocrine. 2018;59(2):319‐329. 28795340 10.1007/s12020-017-1380-8

[53] Ton FN, Gunawardene SC, Lee H, Neer RM. Effects of low-dose prednisone on bone metabolism. J Bone Miner Res. 2005;20(3):464‐470. 15746991 10.1359/JBMR.041125

[54] Kauh E, Mixson L, Malice MP, et al Prednisone affects inflammation, glucose tolerance, and bone turnover within hours of treatment in healthy individuals. Eur J Endocrinol. 2012;166(3):459‐467. 22180452 10.1530/EJE-11-0751

[55] Fleishaker DL, Mukherjee A, Whaley FS, Daniel S, Zeiher BG. Safety and pharmacodynamic dose response of short-term prednisone in healthy adult subjects: a dose ranging, randomized, placebo-controlled, crossover study. BMC Musculoskelet Disord. 2016;17(1):293. 27424036 10.1186/s12891-016-1135-3 PMC4947329

[56] Santiago RA, Silva CA, Caparbo VF, Sallum AM, Pereira RM. Bone mineral apparent density in juvenile dermatomyositis: the role of lean body mass and glucocorticoid use. Scand J Rheumatol. 2008;37(1):40‐47. 18189194 10.1080/03009740701687226

[57] Natsui K, Tanaka K, Suda M, et al High-dose glucocorticoid treatment induces rapid loss of trabecular bone mineral density and lean body mass. Osteoporos Int. 2006;17(1):105‐108. 15886861 10.1007/s00198-005-1923-3

[58] Sharma M, Dhakad U, Wakhlu A, Bhadu D, Dutta D, Das SK. Lean mass and disease activity are the best predictors of bone mineral loss in the premenopausal women with rheumatoid arthritis. Indian J Endocrinol Metab. 2018;22(2):236‐243. 29911038 10.4103/ijem.IJEM_665_17 PMC5972481

[59] Marin RV, Pedrosa MAC, Moreira-Pfrimer LDF, Matsudo SMM, Lazaretti-Castro M. Association between lean mass and handgrip strength with bone mineral density in physically active postmenopausal women. J Clin Densitom. 2010;13(1):96‐101. 20171571 10.1016/j.jocd.2009.12.001

[60] Gibbs JC, Giangregorio LM, Wong AKO, Josse RG, Cheung AM. Appendicular and whole body lean mass outcomes are associated with finite element analysis-derived bone strength at the distal radius and tibia in adults aged 40years and older. Bone. 2017;103:47‐54. 28614701 10.1016/j.bone.2017.06.006

[61] Gregori G, Paudyal A, Barnouin Y, et al Indices of sarcopenic obesity are important predictors of finite element analysis-derived bone strength in older adults with obesity. Front Endocrinol (Lausanne). 2023;14:1279321. 38027147 10.3389/fendo.2023.1279321 PMC10660264

[62] Kanda F, Okuda S, Matsushita T, Takatani K, Kimura KI, Chihara K. Steroid myopathy: pathogenesis and effects of growth hormone and insulin-like growth factor-I administration. Horm Res. 2001;56(Suppl 1):24‐28. 11786681 10.1159/000048130

[63] Hosoyama T, Tachi C, Yamanouchi K, Nishihara M. Long term adrenal insufficiency induces skeletal muscle atrophy and increases the serum levels of active form myostatin in rat serum. Zoolog Sci. 2005;22(2):229‐236. 15738643 10.2108/zsj.22.229

[64] Buckley L, Humphrey MB. Glucocorticoid-induced osteoporosis. N Engl J Med. 2018;379(26):2547‐2556. 30586507 10.1056/NEJMcp1800214

[65] Salcuni AS, Palmieri S, Carnevale V, et al Bone involvement in aldosteronism. J Bone Miner Res. 2012;27(10):2217‐2222. 22589146 10.1002/jbmr.1660

[66] Petramala L, Zinnamosca L, Settevendemmie A, et al Bone and mineral metabolism in patients with primary aldosteronism. Int J Endocrinol. 2014;2014:836529. 24864141 10.1155/2014/836529 PMC4016829

